# Metformin for endothelial dysfunction in non-diabetic disorders: a scoping review

**DOI:** 10.1136/bmjopen-2025-100017

**Published:** 2025-10-06

**Authors:** Roland van Rensburg, Anel Schoonees, Mohammed W Ali, Gert U Van Zyl, Eric H Decloedt

**Affiliations:** 1 Division of Clinical Pharmacology, Department of Medicine, Stellenbosch University Faculty of Medicine and Health Sciences, Cape Town, South Africa; 2 Centre for Evidence-Based Health Care, Department of Global Health, Stellenbosch University Faculty of Medicine and Health Sciences, Cape Town, South Africa; 3 Division of Medical Virology, Department of Pathology, Stellenbosch University Faculty of Medicine and Health Sciences, Cape Town, South Africa

**Keywords:** Review, VASCULAR MEDICINE, HIV & AIDS, CLINICAL PHARMACOLOGY, Drug Utilization, Medicine

## Abstract

**Abstract:**

**Objectives:**

The glucose-lowering drug metformin has shown promise in non-diabetic conditions for improving endothelial dysfunction, but the literature of metformin’s effect on endothelial dysfunction and the biomarkers used to measure endothelial dysfunction have not yet been synthesised.

We aimed to map the extent and nature of the existing research related to metformin for endothelial dysfunction in non-diabetic non-communicable diseases (NCDs). This scoping review was conducted following the methodological framework by Arksey and O’Malley and the recommendations from the Joanna Briggs Institute, and was reported in accordance with the Preferred Reporting Items for Systematic Reviews and Meta-Analyses extension for Scoping Reviews.

**Eligibility criteria:**

We considered any peer-reviewed studies in adult humans on the use of metformin for endothelial dysfunction in non-diabetic NCDs. Narrative reviews, expert opinion, preclinical studies and qualitative studies were excluded.

**Sources of evidence:**

An unrestricted search was conducted on four electronic databases and three registries from inception to October 2024.

**Charting methods:**

Data charting was performed using predetermined data extraction headings. We used a systematic charting method and narrative synthesis to organise, synthesise and report the data.

**Results:**

We identified 56 studies comprising 4620 participants (71.7% female). Polycystic ovarian syndrome was the most investigated NCD (57.1% of studies). 19 distinct biomarkers of endothelial dysfunction were identified, with flow-mediated dilation being the most frequently assessed (18 studies, 745 participants). Metformin showed a trend towards improvement for 7/19 (36.8%) biomarkers. Male participants were underrepresented in the literature and only five studies (9%) were conducted in the global south, potentially limiting the generalisability of repurposed metformin in diverse populations or settings. Studies with an active comparator reported a significant difference between the metformin and comparator groups in 20% (4/20), in contrast to studies without an active comparator (placebo or pre–post studies) reporting significant results favouring metformin in 83.3% (30/36). A knowledge gap also exists for metformin use in people with HIV, given that they are known to develop cardiovascular NCDs at a twofold higher rate than their HIV-negative counterparts.

**Conclusions:**

While there is a growing evidence base supporting metformin as treatment for endothelial dysfunction in non-diabetic NCDs, our scoping review highlighted knowledge gaps in optimal biomarker selection and dosing strategies, and applications in a broader range of NCDs, including in people with HIV. More primary and secondary research using robust methodologies and study designs is needed to determine the quantitative effect of metformin on endothelial dysfunction.

**Strengths and limitations of this study**

- To our knowledge, this is the first study to synthesise the evidence for metformin for endothelial dysfunction in non-diabetic non-communicable diseases (NCDs), guided by the Preferred Reporting Items for Systematic Reviews and Meta-Analyses extension for scoping reviews guideline.
- The broad search terms and eligibility criteria used to identify potentially eligible studies increased the robustness of the scoping review.
- An updated search prior to publication strengthened the completeness of the available data.
- While a wide range of NCDs and biomarkers of endothelial dysfunction were identified, knowledge gaps still exist for metformin use in men, non-endocrine–metabolic NCDs and chronic infectious diseases, such as HIV.
- Further discussions and research are needed to reach consensus on the most optimal biomarkers for endothelial dysfunction.

## Background

 Non-communicable diseases (NCDs) are the current leading cause of mortality globally with 41 million deaths each year, equivalent to 74% of all deaths worldwide.^1^ The top four NCDs, which collectively account for more than 80% of all premature NCD-related deaths, are cardiovascular diseases (CVDs; 17.9 million deaths annually), cancers (9.3 million), chronic respiratory diseases (4.1 million) and diabetes (2.0 million).^1^ CVD alone is the single greatest contributor and accounts for 44% of all NCD-related deaths, and 34% of premature deaths.^2^ The burden of NCDs in low- and middle-income countries (LMICs) is particularly pronounced, where 77% of all deaths and 86% of all premature deaths are NCD related.^1^

The pathogenesis of different NCDs is considered a multifactorial interplay of genetic, physiological, environmental and behavioural factors,^1 3^ but endothelial dysfunction is a common pathway in the development and perpetuation of most NCDs.^4 5^ The endothelium functions as a dynamic and interactive barrier of the innermost lining of the arterial and venous vasculature and controls several complex homeostatic processes designed to nourish and protect surrounding tissues. Consequently, the scientific understanding of the endothelium’s function has evolved from a mere semipermeable biomechanical barrier to an organ that regulates vascular tone and health, cell behaviour, innate immunity, cell–cell interactions and cell metabolism in the vessel wall.^4 6^ Disruption of these functions is integral to the development of not only overt cardiovascular NCDs, such as atherosclerosis, coronary artery disease, hypertension, stroke and peripheral vascular disease, but also has been implicated in the development of pre-eclampsia, diabetes and chronic kidney disease.^5^ The pathogenesis of endothelial dysfunction appears to be a complex interaction between factors largely affecting the dynamics of vasodilation and vasoconstriction, and includes oxidative stress, lipid products, altered blood flow and inflammation.^4^ Many of these factors create an iterative cycle of deteriorating vascular function, and endothelial dysfunction can, therefore, be an initiator and important contributor to the progression of NCDs, such as polycystic ovarian syndrome (PCOS) and metabolic syndrome.^6^ Viral infections, such as hepatitis C, COVID-19 and HIV, are increasingly being recognised as an important risk factor for endothelial dysfunction.^7^,^9^ People living with HIV (PWH) in particular are experiencing a rapidly increasing incidence of HIV-associated NCDs, while HIV-associated communicable diseases decreased significantly due to the effective suppression of viral replication by ART (antiretroviral therapy).^10 11^ A large meta-analysis showed that the global burden of HIV-associated CVD alone tripled over the last 20 years, with the highest burden in sub-Saharan Africa.^12^ The mechanism underlying this increase in CVD has been strongly linked to HIV-associated inflammation and subsequent endothelial dysfunction.^7^

Given the dynamic nature of the endothelium, endothelial dysfunction has emerged as a viable modifiable target, given the pervasive prevalence of NCDs and the role that endothelial dysfunction plays in their pathogenesis and perpetuation. Consequently, several therapeutic interventions have been investigated to treat or prevent endothelial dysfunction. These include lipid-lowering drugs, antihypertensive drugs, antidiabetic drugs, anti-inflammatory drugs, antiplatelet drugs, disease-modifying antirheumatic drugs, vitamin C and E, N-acetylcysteine and several other experimental therapies.^413^,^16^ Much of the evidence for these interventions is from preclinical studies or with methodological shortcomings in human trials that limit implementation, such as small sample sizes and uncontrolled study groups.

Metformin has gained increasing attention as a repurposed drug for various aspects of NCDs, including weight loss, immune modulation, gut microbiome modulation and, in particular, improvement of endothelial dysfunction.^17^,^23^ Metformin is a well-known, affordable and widely available glucose-modulating drug used in type 2 diabetes mellitus. Metformin’s mechanism of action involves sensitisation of tissues to circulating insulin, but rarely results in hypoglycaemia when used as monotherapy at therapeutic doses.^24^ As such, metformin is considered safe, even in normoglycaemic patients.^25^

In diabetic patients, metformin’s direct effect on endothelial dysfunction was shown in a randomised controlled trial where the improvements seen in soluble biomarkers of endothelial dysfunction were generally independent of metformin-associated changes in glycaemic control and weight.^26^ In recent years, however, its beneficial effects on endothelial function in non-diabetic patients have been actively investigated.^6 18^ Metformin has been shown to significantly improve endothelial dysfunction, independent of its glucose-lowering mechanism.^17^ Illustratively, in a study of 172 propensity score-matched participants with prediabetes and stable angina (with half on metformin), those on metformin had less coronary artery endothelial dysfunction as assessed by an acetylcholine infusion than those not on metformin (p<0.05).^27^ This beneficial outcome is postulated to be linked to metformin’s pleiotropic effects to modulate molecular mechanisms, including the reduction of oxidative stress and proinflammatory cytokines, such as tumour necrosis factor alpha and interleukin 6, and the normalisation of anti-inflammatory biomarkers, such as adiponectin and sirtuin-6.^17 28 29^ Additionally, data from both preclinical models and clinical studies of diabetic and non-diabetic patients support that metformin should be further investigated for its beneficial effects in improving endothelial and cardiovascular function, longevity and overall health.^6 17^

Despite growing evidence of metformin’s potential for treating endothelial dysfunction in non-diabetic NCDs, the literature on the methods to determine endothelial dysfunction and the effect of metformin to improve this is currently lacking. The overarching aim of this scoping review is to map the extent and nature of existing research related to metformin for endothelial dysfunction in non-diabetic NCDs, specifically the range of different NCDs, the biomarkers used for determining endothelial dysfunction and response to interventions and the formulations and doses of metformin studied. The secondary aims are to quantify the effect of metformin according to the different biomarkers of endothelial dysfunction and to identify gaps and areas for further research.

## Methods

The scoping review was conducted based on the methodological framework of Arksey and O’Malley as well as the latest guidance from the Johanna Briggs Institute.^30 31^ The protocol was prospectively registered (https://osf.io/98aru/). Reporting was done according to the Preferred Reporting Items for Systematic Reviews and Meta-Analyses Extension for Scoping Reviews checklist (online supplemental file 1).^32^

### Study objectives

The scoping review sought to answer the research question: ‘In patients without diabetes, what is the utility of metformin for endothelial dysfunction?’

The objectives of the review were to scope the existing body of literature to determine the following.

1. For which endothelial disorders has metformin been used as an intervention?
2. What are the metformin formulations and dose ranges used for endothelial dysfunction?
3. To what degree has metformin improved endothelial dysfunction?

### Eligibility criteria

#### Population

Any literature on the use of metformin for endothelial dysfunction in humans was considered. Adults by any definition (as defined by study authors) were included. Participants with any type of diabetes mellitus were excluded, but participants with pre-diabetes or insulin resistance were included, as the effect of subclinical degrees of hyperglycaemia on endothelial dysfunction is less well known. No other limitations based on population characteristics (including sex/gender or race/ethnicity) were applied.

#### Concept

All studies containing any information on metformin as an intervention for endothelial dysfunction as an outcome were included. Studies where metformin was the sole intervention compared with a control group were preferred, where multiple interventions were involved, and comparison groups should have received the same treatment apart from the administration of metformin.

Methods to determine endothelial dysfunction were defined as those biomarkers directly attributable to the endothelium, including blood biomarkers, flow-mediated dilation (FMD), including peripheral artery tonometry, and direct imaging with or without pharmacological aid.

#### Context

All sources of evidence pertaining to any contextual setting were eligible for inclusion.

### Evidence sources

We included as eligible any type of peer-reviewed primary interventional study or systematic review on metformin for endothelial dysfunction in non-diabetic patients. Primary studies included controlled and uncontrolled randomised controlled trials, and pre–post (before-and-after) studies. Systematic reviews were required to have clear objectives, clear criteria for inclusion, searched at least two electronic databases and performed data extraction and risk of bias assessment. If a systematic review included the primary studies that were also identified in the search, the primary studies were used and the systematic review’s reference list was checked to ensure that all eligible studies were included. Observational studies, such as cohort studies, case–control studies, cross-sectional studies, case series and case reports, were considered eligible for inclusion. We also included eligible studies that were registered or ongoing if the protocol was published and met the inclusion criteria. When the protocol registry indicated that the study had been completed, an individual search for a published manuscript was conducted, or the study investigator(s) were contacted for the results. When the search identified only the abstract of a study, the full text was preferentially sought. Literature (or narrative) reviews and other forms of expert opinion were excluded, as well as qualitative and preclinical studies. As this was a scoping review of published data, patients and/or the public were not involved in the design, conduct, reporting, or dissemination plans of this research.

### Search strategy

A systematic search of the available literature was conducted in August 2023 by an information specialist (AS). No language, date or publication restrictions were applied, and studies investigating diabetes were not excluded from the terms to ensure a comprehensive search. The search strategy was peer reviewed according to the PRESS checklist.^33^ Four electronic databases were searched: Medline (PubMed), the Cochrane Library (specifically the Cochrane Database of Systematic Reviews (CDSR) and CENTRAL), Web of Science Core Collection (Clarivate Analytics) and Epistemonikos (https://www.epistemonikos.org/). The search was also conducted in three registries: PROSPERO register (National Institute for Health and Care Research; https://www.crd.york.ac.uk/PROSPERO/), Clinicaltrials.gov (https://clinicaltrials.gov/) and the WHO’s International Clinical Trials Registry Platform (https://trialsearch.who.int/). Due to the diverse scope of the research question, other forms of grey literature were not searched. The full search strategy can be found in online supplemental file 2.

### Data charting

Data to be extracted were discussed by the reviewers a priori to ensure information integrity, completeness and uniformity. A data extraction sheet was developed in Microsoft Excel and field tested with a randomly selected study. Following the comprehensive search, the results were uploaded to the research synthesis software Rayyan (https://www.rayyan.ai), and a single reviewer (RvR) screened through titles and abstracts to identify potentially eligible studies and reviews. Due to the single-reviewer approach, the screening favoured overinclusion rather than underinclusion so that the full texts could be more extensively interrogated by two reviewers. Full texts of potentially eligible studies were obtained and independently assessed for inclusion by two reviewers (RvR and MWA). Reference lists of key articles identified in the primary search were explored to identify additional relevant evidence that may have been missed during the initial database search. Uncertainty regarding eligibility during the selection process was resolved by discussion or by consultation with a third reviewer (AS) where required. Extracted data included the study first author, year of publication, study design, country(ies) of study, sample size, participant characteristics, the NCD investigated, details of metformin, details of the comparator(s), endothelial dysfunction biomarkers and the main findings relating to endothelial dysfunction. Where a study was eligible on full-text screening, but vital data were missing (such as details of participants, comparators or outcomes), the study author(s) were contacted. When no response was received, a second request was sent. If no response was received after the second request, the study was excluded.

### Quality appraisal

This scoping review aimed to map out and identify gaps in the existing evidence. As such, methodological quality assessment of the included studies was not done.

### Data analysis

The extracted data were categorised according to the NCD under investigation. Data were tabulated according to the predetermined data charting headings, and summary statistics were calculated. Our protocol methods did not include quality appraisal to allow meta-analytic pooling of the endothelial dysfunction outcomes to determine the overall effect size of metformin compared with other interventions. However, in line with our third objective, we determined the individual study effect sizes of the mean change in the biomarkers between metformin and the comparators where possible to visually display the trends, using the methodology described in the Cochrane Handbook for Systematic Reviews of Interventions.^34^ Accordingly, where the SD of the change between groups was specified, we used this SD to calculate the correlation coefficient for the studies of the same endothelial dysfunction biomarker that did not report the SD of the change. Where no correlation coefficient could be calculated from at least one study in a particular biomarker group, the SD of the change was calculated using an empiric correlation coefficient of 0.5 as a conservative value.^34^ The mean changes and their associated SD for metformin and the comparator for each endothelial dysfunction biomarker were compared using a random effects model and presented graphically as a visual representation of the effect trends using STATA (release 18.5, StataCorp LLC, College Station, Texas, USA). The overall effect size and heterogeneity statistics were not reported to avoid interpretation as a meta-analysis.

## Results

### Selection of included studies

**Figure 1:**
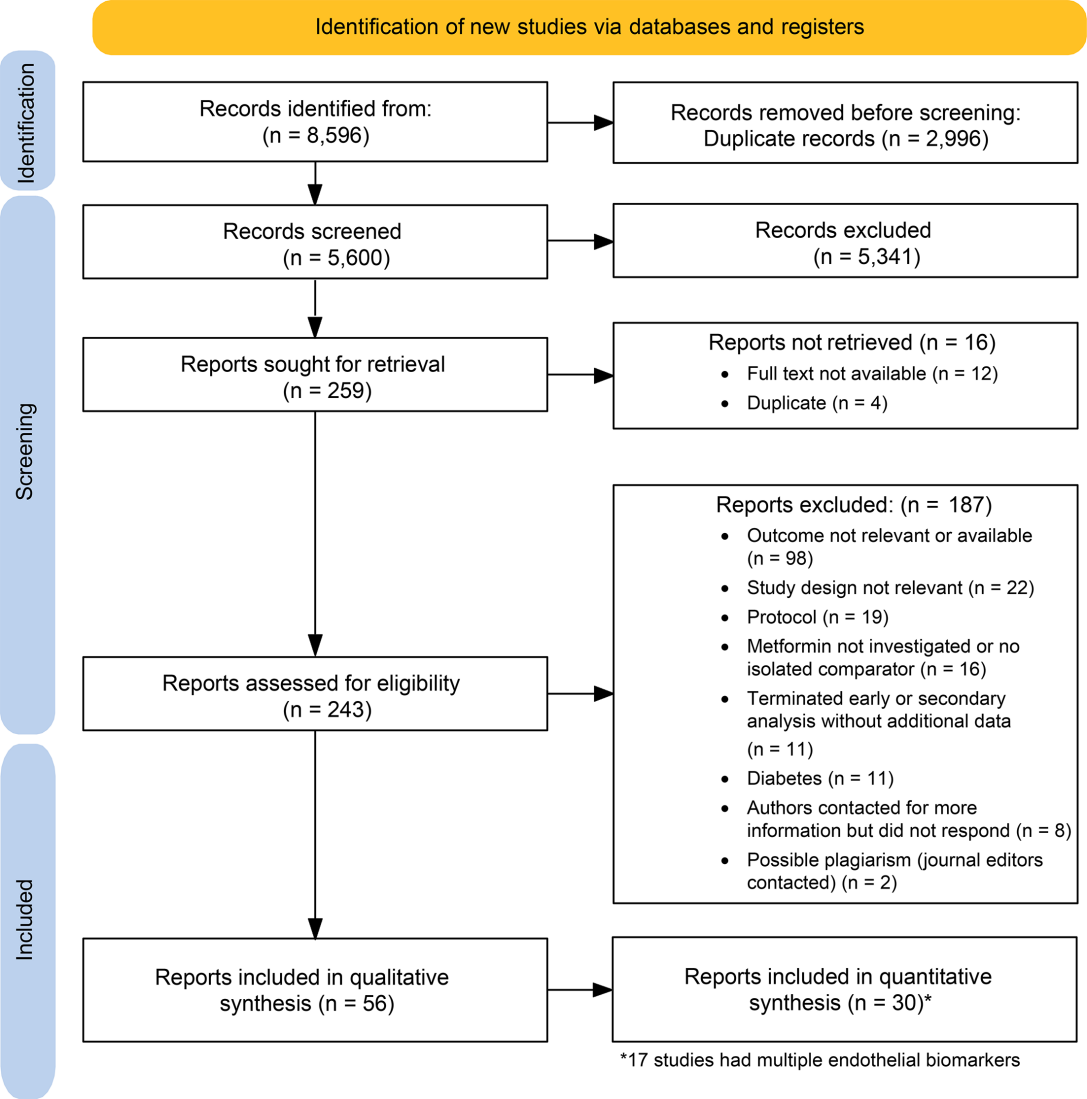
Overview of the selection process and reasons for exclusion.

Our comprehensive search yielded 8596 results. After removing 2996 duplicates, 5600 records were screened by title and abstract. Of these, 259 were full-text reviewed, and 56 records were included in the final research synthesis (figure 1). The data of the included records were assessed for suitability for the quantitative effect size analysis, and 30 records representing 19 endothelial dysfunction biomarkers were included. The search was conducted again on 30 October 2024 with the same search terms from August 2023 to identify any new evidence. Of the 291 new reports found, 3 new eligible systematic reviews were identified, but no new eligible primary studies among the search yield. Additionally, after checking the reference lists of the systematic reviews, no new primary studies were identified. The updated PRIMSA flowchart is shown in online supplemental file 3.

### Study and participant characteristics

The 56 included studies embodied 4620 participants. Details of the included studies are tabled in online supplemental file 4. Table 1 summarises the participant characteristics, showing on average a middle-aged, overweight population. Most were female (71.7%), likely due to the large representation of PCOS studies (n=32/56, 57.1%). 57% of the studies (32/56) were conducted as randomised, controlled, parallel design trials, totalling 3903 participants (84.5%) (table 2). The included studies represented a wide range of global regions, with the USA being the most common study country with 2211 participants across 8 studies. The earliest study was published in 1984, and the most recent one in 2023. The most common study decade was between 2010 and 2019, when 27 studies were conducted in 3286 participants (71.1%).

**Table 1:** Participant characteristics

Participant characteristics	Metformin group*	Comparator group*†
Female sex, n/N (%) Included studies n (%)‡	3314/4620 (71.7%) n=54 (94.7%)	
Age, mean (SD) Included studies n (%)‡	40.0 (16.2) n=48 (84.2%)	44.5 (15.5) n=29 (50.9%)
Weight, mean (SD) Included studies n (%)‡	79.1 (21.5) n=20 (35.1%)	77.9 (21.8) n=15 (26.3%)
BMI, mean (SD) Included studies n (%)‡	29.7 (5.9) n=50 (87.7%)	29.7 (5.7) n=28 (49.1%)

* Data reporting 95% CIs or median and IQR were converted to mean and SD using the approaches of Wan *et al*^45^ and the Cochrane Handbook.^46^

† Comparator group included less studies due to the removal of single intervention pre–post studies (n=38).

‡ Studies were included where comparable data were available. Percentage expressed at percentage of total (N=56).

BMI, body mass index.

**Table 2:** Study characteristics

		Number of studies (N=56)	Number of participants (N=4620)
**Study design**			
Uncontrolled experimental trial (pre–post metformin assessment)		19	502
Parallel trial	Randomised, controlled, open label	17	846
	Randomised, controlled, double blind	15	3057
Crossover trial	Randomised, controlled, open label	2	71
	Randomised, controlled, double blind	2	24
Experimental (assignment not stated)		1	120
**Country**			
USA		8	2211
Italy		6	159
Poland		5	324
China		4	374
Scotland		4	331
UK*		4	126
Türkiye		4	121
Greece		4	91
Mexico		2	94
Spain		2	67
Brazil		2	61
Germany		2	42
Venezuela		2	31
France		1	324
Australia		1	67
Iran		1	60
Iraq		1	54
Netherlands		1	39
Slovenia		1	26
Israel		1	18
**Publication year**			
1980–1989		1	13
1990–1999		2	340
2000–2009		21	707
2010–2019		27	3286
2020–2023		5	274

* Involving multiple UK countries.

UK, United Kingdom; USA, United States of America.

Our search found two published studies on metformin in non-diabetic PWH (Van Wijk, *et al*^35^ and Fitch, *et al*^36^). Both were randomised, controlled, open-label trials, and the NCDs assessed were HIV lipodystrophy and atherosclerosis in PWH. The viral and ART characteristics are presented in online supplemental file 5.

No systematic reviews were included, as all eligible studies in the reference lists of the systematic reviews were already included as primary studies according to our methodology. Additionally, the identified systematic reviews’ research questions were not aligned with our exploratory research question.

### NCDs and biomarkers of endothelial dysfunction

**Figure 2:**
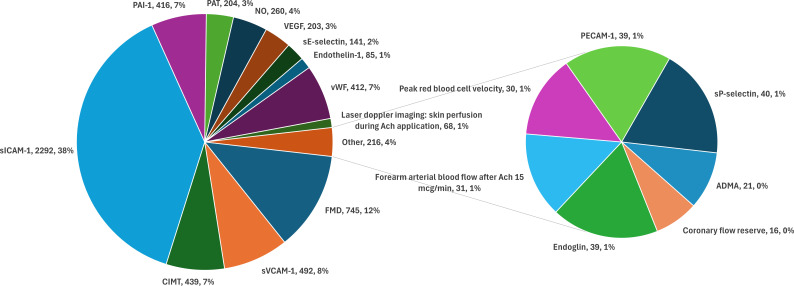
Biomarkers of endothelial dysfunction (labelled as biomarker, number of participants and percentage of total). Ach, acetylcholine; CIMT, carotid intima–media thickness; FMD, flow-mediated dilation; NO, nitric oxide; PAI-1, plasminogen activator inhibitor-1; PAT, peripheral arterial tonometry; PECAM-1, platelet endothelial cell adhesion molecule-1; sE-selectin, serum E-selectin; sICAM-1, soluble intercellular adhesion molecule-1; sP-selectin, serum P-selectin; sVCAM-1, soluble vascular cell adhesion molecule-1; VEGF, vascular endothelial growth factor; vWF, von Willebrand factor.

Metabolic-related disorders were the most common NCDs investigated, with the top 4 being PCOS (57.1%), metabolic syndrome (14.3%), insulin resistance/impaired glucose tolerance (7.1%) and overweight/obesity (7.1%), accounting for 88.6% of all participants (n=4093/4620, table 3). Our review found 19 distinct biomarkers for endothelial dysfunction (figure 2). PCOS had the widest range of endothelial dysfunction biomarkers, totalling 16 biomarkers across 54 studies. FMD was the most common biomarker assessed (18 studies involving 745 participants), followed by soluble vascular cell adhesion molecule-1 (sVCAM-1, 10 studies involving 492 participants) and carotid intima–media thickness (CIMT, 9 studies involving 439 participants). The number of participants linked to each biomarker across the NCDs investigated is displayed in the interactive Sankey diagram in online supplemental file 6.

**Table 3:** NCDs investigated

	Number of studies (N=56)	Number of participants (N=4620)
**NCD assessed**		
PCOS	32	1121
Metabolic syndrome	8	317
Insulin resistance/impaired glucose tolerance	4	2119
Overweight/obesity	4	536
Coronary artery disease	3	269
Early endometrial adenocarcinoma	1	120
Chronic heart failure	1	62
HIV lipodystrophy	1	39
Atherosclerosis in PWH	1	24
Peripheral vascular disease	1	13

NCD, non-communicable disease; PCOS, polycystic ovarian syndrome; PWH, people living with HIV.

### Metformin and other interventions

93% of the studies reported using the immediate-release metformin formulation at a median total daily dose of 1.7 g (IQR 1.5–1.7, table 4). The remaining 7% used the extended-release formulation at a median total daily dose of 1.6 g (IQR 1.5–1.7). No comparator was used in 37.5% (n=20/56) of studies (pre–post metformin assessment), with placebo as the comparator accounting for 26.8% (n=15/56) of studies. Hormonal contraceptives were the most common active comparator, used in 12.5% (n=7/56) of studies. Overall, the median duration of the studies was 4 months (IQR 3–6, table 5). The longest duration was 12 months in three studies evaluating coronary artery disease and one study on atherosclerosis in PWH.

**Table 4:** Intervention characteristics

INTERVENTIONS	Number of studies (N=56)	Percentage of studies (%)	Total dose/day
**Metformin**			
IR formulation	52	93	1.7 g (1.5–1.7) (0.3–3)*
XR formulation	4	7	1.6 g (1.5–1.7) (1.5–2)*
**Comparator**			
No comparator (pre–post metformin assessment)	20	35.7	–
Placebo	15	26.8	–
Ethinylestradiol and cyproterone acetate	4	7	35 mcg 2 mg
Rosiglitazone	3	5.4	4 mg (4–8)†
Simvastatin	2	3.6	20 mg
Atorvastatin	1	1.79	20 mg
Observational control group receiving no drug or placebo	1	1.79	–
Empagliflozin	1	1.79	25 mg
Ethinylestradiol and drosperinone	1	1.79	30 mcg 3 mg
Ethinylestradiol and norgestimate	1	1.79	35 mcg 0.18/0.215/0.25 mg
Exenatide	1	1.79	10 mcg
Fenofibrate	1	1.79	200 mg
Intensive lifestyle modification	1	1.79	three supervised exercise sessions/week and dietary counselling one time/week
Medroxyprogesterone acetate	1	1.79	400–800 mg†
Myoinositol and folic acid	1	1.79	4 g 400 mcg
Pioglitazone	1	1.79	30 mg
Rimonabant	1	1.79	20 mg

* Median (IQR) (range).

† Range.

IR, immediate release; XR, extended release.

**Table 5:** Study durations

Study duration	Number of studies (N=56)	Duration, months*
**Overall**	55†	4 (3–6) (1.5–18)
**By NCD**		
PCOS	32	5 (3–6) (2–6)
Metabolic syndrome	8	3 (3–4) (1.5–12)
Insulin resistance/impaired glucose tolerance	4	4 (3–8.5) (3–12)
Overweight/obesity	3†	3 (2–12) (2–12)
Coronary artery disease	3	12 (2–18) (2–18)
Early endometrial adenocarcinoma	1	4
Chronic heart failure	1	4
HIV lipodystrophy	1	6.5
Atherosclerosis in PWH	1	12
Peripheral vascular disease	1	6

* Median (IQR) (range).

† N=55 (one study excluded due to pregnancy duration determining study duration).

NCD, non-communicable disease; PCOS, polycystic ovarian syndrome; PWH, people living with HIV.

### Effect of metformin on endothelial dysfunction

**Figure 3:**
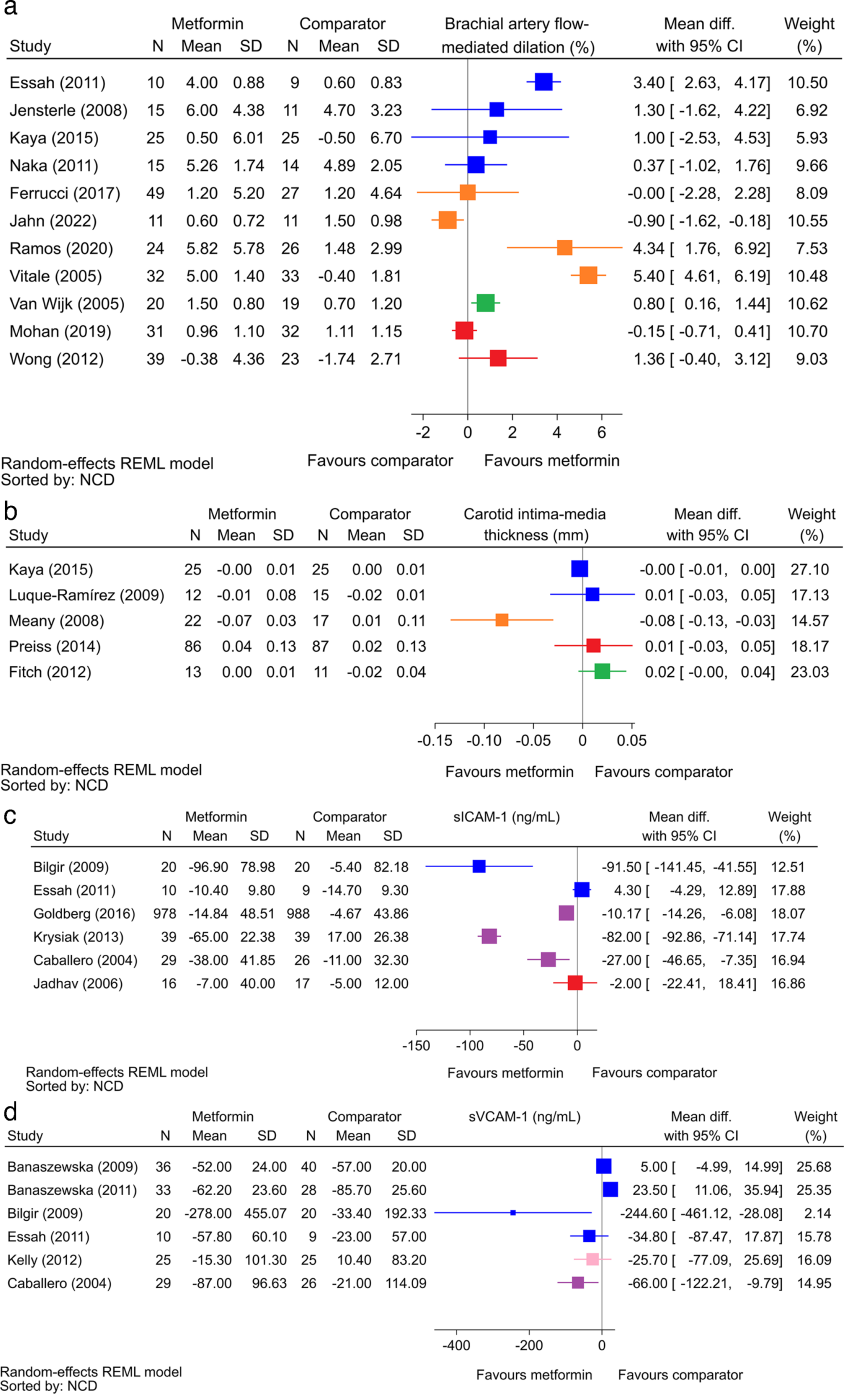
Top four endothelial biomarkers with most included studies showing the mean difference in effect between metformin and the comparator for (**a**) brachial artery FMD (colour key: blue—PCOS; orange—metabolic syndrome; green—HIV and red—cardiovascular). (**b**) CIMT (colour key: blue—PCOS; orange—metabolic syndrome; green—HIV and red—cardiovascular). (**c**) sICAM-1 (colour key: blue—PCOS; purple—impaired glucose tolerance and red—cardiovascular). (**d**) sVCAM-1 (colour key: blue—PCOS; pink—overweight or obesity and purple—impaired glucose tolerance). CIMT, carotid intima–media thickness; FMD, flow-mediated dilation; NCD, non-communicable disease; PCOS, polycystic ovarian syndrome; sICAM-1, soluble intercellular adhesion molecule-1; sVCAM-1, soluble vascular cell adhesion molecule-1.

Figure 3 visually shows the effect of metformin on endothelial dysfunction for the top four biomarkers of the most included studies (additional biomarkers are included in online supplemental file 7). There was a visual trend towards benefit with metformin for the following biomarkers: brachial artery FMD, CIMT, soluble intercellular adhesion molecule-1 (sICAM-1), sVCAM-1, vascular endothelial growth factor (VEGF), nitric oxide (NO) and von Willebrand factor, expressed as a percentage (vWF %). The mean difference showing a trend towards favouring the comparator was found for plasminogen activator inhibitor-1 only and had a neutral trend, favouring neither metformin nor the comparator, for the following biomarkers: peripheral arterial tonometry (PAT), E-selectin and serum E-selectin, endoglin, absolute ICAM-1, absolute VCAM-1, absolute platelet endothelial cell adhesion molecule-1, skin perfusion during acetylcholine application using laser Doppler imaging, serum P-selectin and vWF expressed in IU/dL and U/L. When the top four biomarkers of the most included studies were stratified by the NCDs that investigated those biomarkers (figure 3), PCOS, metabolic syndrome and impaired glucose tolerance showed a trend towards benefit across the biomarkers, whereas HIV, cardiovascular NCDs and overweight or obesity showed an equipoise trend.

## Discussion

Our scoping review identified 56 primary studies of metformin for endothelial dysfunction in non-diabetic NCDs. Overall, metformin was broadly investigated in several NCDs, with endocrine–metabolic disorders—in particular PCOS—being the most common indication. Brachial artery FMD, CIMT, sICAM-1 and sVCAM-1 were the four most commonly investigated biomarkers, with endocrine–metabolic disorders showing the most trends for benefit with metformin.

### Population and studies

The global interest in metformin as a repurposed intervention beyond diabetes is reflected in our finding of the increasing number of publications and participants since the 2000s. Most studies investigated metformin for PCOS, and male participants are underrepresented in the current literature (28.3% of participants). Most studies had small sample sizes, with a median of 34 participants per study (IQR 20–61). Furthermore, only five of the studies (9%) were conducted in the global south, potentially limiting the generalisability of the repurposed metformin in diverse populations or settings. Treatment allocation was not blinded in 38/56 (68%) of the studies, either as pre–post or open-label studies, potentially introducing selection and/or detection bias. However, as most outcomes were blood biomarkers, the risk of detection bias is likely minimised.

There exists a paucity of data in the current literature of metformin for chronic infectious disease-related endothelial dysfunction. Our search identified only HIV-associated NCDs, likely due to modern curative strategies associated with hepatitis C, and the generally short duration and novelty of COVID-19. The dearth of metformin for HIV-associated NCDs represents a major gap in the literature, especially given the twofold higher rate of CVD-linked NCDs in PWH compared with HIV-negative counterparts.^10^,^12^ The two studies in PWH that were included in this scoping review also used older ART regimens (protease inhibitor- and non-nucleoside reverse transcriptase inhibitor-based regimens), and the findings may, therefore, not be generalisable to newer integrase strand transfer inhibitor-based regimens. Additionally, effective interventions for CVD in PWH are often unaffordable on a larger scale or unavailable in LMICs. Illustratively, the large, randomised REPRIEVE trial that investigated the statin pitavastatin for the prevention of major CVD in PWH showed a significant reduction of 35% in major CVD events over the median follow-up of 5.1 years compared with placebo.^37^ However, pitavastatin is not readily available in most LMICs where the overlapping burdens of HIV and NCDs are highest, and the effect cannot be confidently extrapolated to other, readily available statins. Additionally, the comparatively high cost of generic pitavastatin to other generic statins is prohibitive in most LMICs.^38^ Therefore, given the rapid increase in NCDs, especially in LMICs, accessible, affordable and safe drugs are needed to address endothelial dysfunction in most of the world’s underserved population.^39^

### Metformin dose

Metformin has a wide dosing range, from 500 mg to 3000 mg per day.^40^ The reported median daily dose of both the immediate-release formulation (1.7 g) and the extended-release formulation (1.6 g) of metformin in this review is, therefore, considered to be in midrange of dosing with the IQR 1.5–1.7 g per day. While a clear dose–response relationship of metformin with glycaemic control has been shown with type 2 diabetes,^41 42^ this review highlights the current uncertainty in the literature of whether higher doses in non-diabetic endothelial disorders will confer greater benefit on endothelial biomarkers, given that a dose–response relationship of metformin’s pleiotropic effects has not yet been confirmed. A lack of a clear dose–response relationship was shown in a multicentre, prospective cohort study in PCOS, where, after 6 months, the clinical and endocrine–metabolic features did not differ significantly among metformin daily doses of 1000 mg, 1500 mg and 1700 mg.^43^

### Comparator interventions

Of the reported studies, 64.3% (36/56) did not have an active comparator, which included 35.7% (20/56) that used the same participants as internal controls (pre–post metformin assessment). The paucity of an independent, external comparator group in these studies may have introduced confounding, limiting the reliability and generalisability of the results. Illustratively, of the studies with an active comparator, the authors reported a significant difference between the metformin and comparator groups in the biomarker outcomes in only 20% (4/20) of the studies. In comparison, the studies without an active comparator (placebo or pre–post metformin assessment) reported significant results favouring metformin in 83.3% (30/36) of studies, accentuating potential bias and confounding.

### Biomarker outcomes

Our scoping review highlighted the wide range of biomarkers investigated for endothelial dysfunction, with a trend for benefit with metformin found with direct measurement biomarkers (brachial artery FMD and CIMT) and soluble biomarkers (sICAM-1, sVCAM-1, VEGF, NO and vWF %). The diversity is expected, as a robust and reliable biomarker for endothelial dysfunction that can be applied across a range of conditions has not yet been determined. Of the 19 biomarkers investigated, 36.8% (7/19) were quantified using imaging or plethysmography: FMD, CIMT, PAT, skin perfusion using laser Doppler imaging, coronary flow reserve, forearm arterial blood flow and peak red blood cell velocity. The remaining 63.2% (12/19) were soluble biomarkers in peripheral blood. It can be reasonably argued that the direct measurements of the endothelium using imaging or plethysmography are more specific for endothelial dysfunction, as opposed to blood biomarkers that can be influenced by non-endothelial factors. However, three of the direct biomarkers, FMD, CIMT and coronary flow reserve, are user dependent and, therefore, liable to variation based on operator skill and experience.^44^ Of the biomarkers where the difference in effect between metformin and the comparator could be determined, a visual trend towards benefit with metformin was noted with the user-dependent biomarkers FMD and CIMT, whereas the trend was neutral for the user-independent biomarkers PAT and skin perfusion using laser Doppler imaging. Accordingly, the assessment of user-dependent biomarkers may introduce observer and measurement bias.

The average treatment duration of 4 months in the identified studies may also be too short to confidently assess metformin’s effect on the biomarkers of endothelial dysfunction. This raises the question of whether a longer treatment period may have led to more significant results if a difference in response truly existed. However, the percentage of studies reporting significant effects of metformin on endothelial dysfunction was the same in those with a duration ≤4 months compared with those>4 months: 53.6% (15/28) in each group. Nevertheless, it is still unknown whether metformin treatment beyond the upper limit of the reported study duration of 18 months will confer a differential benefit, especially considering the insidious development of most NCDs.

### Future directions

Longer term studies incorporating larger sample sizes, different metformin doses and preferably active-comparator randomised controlled designs or analytical prospective cohort studies are needed. These approaches will aid in more accurately determining the association of metformin with endothelial dysfunction biomarkers and clinical outcomes. Additionally, further discussions and research are needed to reach consensus on the most optimal biomarkers for endothelial dysfunction. Future research should also determine the pooled quantitative effect of metformin on different biomarkers of endothelial dysfunction using robust quality appraisal methodologies, ideally as systematic reviews and meta-analyses using the trends identified in our findings.

### Limitations

Our approach in conducting the research as a scoping review and not a systematic review with methodological quality appraisal limits the strength of this research to draw conclusions on the effect of metformin on the biomarkers of endothelial dysfunction. These biomarkers are also early indicators of endothelial dysfunction, and due to the available literature, we were not able to assess the long-term utility of metformin on NCD improvement, nor on the prevention of NCD development. While the sparsity of studies investigating metformin for HIV-associated NCDs precluded us from answering this specific aspect of our research question, our review emphasised the need for more research into this large, underserved population who are predominantly situated in LMICs in the global south. Furthermore, the single-reviewer approach to the initial title and abstract screening was not ideal, although the broad criteria applied during this initial, sensitive screening process favoured overinclusion rather than underinclusion, thereby mitigating the risk of unduly excluded studies. Finally, grey literature was excluded from the search and could have contributed to publication bias; however, the review aimed to map peer-reviewed and indexed evidence to ensure replicability and methodological transparency.

## Conclusion

Our scoping review provided a comprehensive and encompassing picture of the peer-reviewed literature on the utility of metformin for endothelial dysfunction in non-diabetic NCDs. The review found that a broad range of endothelial biomarkers, most commonly FMD and CIMT, have been investigated as the endpoints of treatment with metformin for several NCDs. The review also highlighted major gaps that still exist in the literature regarding the identification of a robust, ubiquitous biomarker across NCDs, the effect of higher doses and longer durations of metformin and studies investigating HIV-associated NCDs. While the absolute quantitative effect of metformin on the biomarkers of endothelial dysfunction could not be determined, our review highlights potential research opportunities and provides a platform for conducting future high-quality primary research, such as randomised controlled trials or analytical prospective cohort studies, and secondary research, in particular systematic reviews and meta-analyses. Additionally, our review offers preliminary data on the trends in endothelial biomarkers for further investigations on the repurposing of metformin for NCDs.

## Supplementary material

10.1136/bmjopen-2025-100017 online supplemental file 1

10.1136/bmjopen-2025-100017 online supplemental file 2

10.1136/bmjopen-2025-100017 online supplemental file 3

10.1136/bmjopen-2025-100017 online supplemental file 4

10.1136/bmjopen-2025-100017 online supplemental file 5

10.1136/bmjopen-2025-100017 online supplemental file 6

10.1136/bmjopen-2025-100017 online supplemental file 7

## Data Availability

All data relevant to the study are included in the article or uploaded as supplementary information.
