# Supplementary material for: Metformin for endothelial dysfunction in non-diabetic disorders: a scoping review
Source: BMJ Open. 2025 Oct 6;15(10):e100017. doi: 10.1136/bmjopen-2025-100017 (PMC12506206; doi:10.1136/bmjopen-2025-100017)
Supplement: online supplemental file 2 [file bmjopen-15-10-s002.docx]

**Supplementary 2 – Search strategy:**

The following search terms were applied to four electronic databases: Medline (PubMed), the Cochrane Library (specifically the CDSR and CENTRAL), Web of Science Core Collection (Clarivate Analytics), and Epistemonikos (<https://www.epistemonikos.org/>); and three registries: PROSPERO register (National Institute for Health and Care Research; <https://www.crd.york.ac.uk/PROSPERO/>), Clinicaltrials.gov (<https://clinicaltrials.gov/>), and the World Health Organization’s International Clinical Trials Registry Platform (ICTRP, <https://trialsearch.who.int/>).

**PubMed:**

**#1** “Metformin”[Mesh] OR metformin[Title/Abstract]

**#2** endothelium OR endothelial OR vascular OR vasodilation OR vasodilator OR “blood flow” OR “flow mediated dilation” OR FMD OR “FMD-BA” OR ultrasound OR acetylcholine OR “nitric oxide” OR microvascular OR microcirculation OR “peripheral artery tonometry” OR “brachial artery reactivity test” OR BART OR “reactive hyperaemia” OR RH-PAT OR EndoPAT

**#3** #1 AND #2

**#4** “hiv infections”[MeSH Terms] OR “hiv”[MeSH Terms]

**#5** "HIV"[Title/Abstract] OR "hiv-1"[Title/Abstract] OR "hiv 2*"[Title/Abstract] OR "hiv1"[Title/Abstract] OR "hiv2"[Title/Abstract] OR "hiv infect*"[Title/Abstract] OR "human immunodeficiency virus"[Title/Abstract] OR "human immune deficiency virus"[Title/Abstract] OR "human immuno deficiency virus"[Title/Abstract] OR "human immune deficiency virus"[Title/Abstract] OR ("human immun*"[Title/Abstract] AND "deficiency virus"[Title/Abstract]) OR "acquired immunodeficiency syndromes"[Title/Abstract] OR "acquired immune deficiency syndrome"[Title/Abstract] OR "acquired immuno deficiency syndrome"[Title/Abstract] OR "acquired immune deficiency syndrome"[Title/Abstract] OR ("acquired immun*"[Title/Abstract] AND "deficiency syndrome"[Title/Abstract]) OR "hiv aids"[Title/Abstract]

**#6** #4 OR #5

**#7** #3 AND #6

*We ran this search strategy with the two HIV-related search strings above (#4 and #5). However, this limited the search yield to 9 records with 2 eligible studies and we decided to omit these terms, and run with the search with #3 only. The HIV-related search strings were subsequently also omitted from the remaining databases and registries.*

**Cochrane Library:**

**#1** MeSH descriptor: [Metformin] explode all trees

**#2** (metformin):ti,ab,kw

**#3** #1 OR #2

**#4** endothelium OR endothelial OR vascular OR vasodilation OR vasodilator OR “blood flow” OR “flow mediated dilation” OR FMD OR “FMD-BA” OR ultrasound OR acetylcholine OR “nitric oxide” OR microvascular OR microcirculation OR “peripheral artery tonometry” OR “brachial artery reactivity test” OR BART OR “reactive hyperaemia” OR RH-PAT OR EndoPAT

**#5** #3 AND #4

**Web of Science Core Collection:**

(((TS=(metformin)) OR TI=(metformin)) OR AB=(metformin)) AND ALL=(endothelium OR endothelial OR vascular OR vasodilation OR vasodilator OR “blood flow” OR “flow mediated dilation” OR FMD OR “FMD-BA” OR ultrasound OR acetylcholine OR “nitric oxide” OR microvascular OR microcirculation OR “peripheral artery tonometry” OR “brachial artery reactivity test” OR BART OR “reactive hyperaemia” OR RH-PAT OR EndoPAT)

**Epistemonikos:**

(advanced_title_en:((advanced_title_en:(metformin) OR advanced_abstract_en:(metformin))) ORadvanced_abstract_en:((advanced_title_en:(metformin)ORadvanced_abstract_en:(metformin)))) AND (advanced_title_en:((advanced_title_en:(endothelium OR endothelial OR vascular OR vasodilation OR vasodilator OR "blood flow" OR "flow mediated dilation" OR FMD OR "FMD-BA" OR ultrasound OR acetylcholine OR "nitric oxide" OR microvascular OR microcirculation OR "peripheral artery tonometry" OR "brachial artery reactivity test" OR BART OR "reactive hyperaemia" OR RH-PAT OR EndoPAT) OR advanced_abstract_en:(endothelium OR endothelial OR vascular OR vasodilation OR vasodilator OR "blood flow" OR "flow mediated dilation" OR FMD OR "FMD-BA" OR ultrasound OR acetylcholine OR "nitric oxide" OR microvascular OR microcirculation OR "peripheral artery tonometry" OR "brachial artery reactivity test" OR BART OR "reactive hyperaemia" OR RH-PAT OR EndoPAT))) OR advanced_abstract_en:((advanced_title_en:(endothelium OR endothelial OR vascular OR vasodilation OR vasodilator OR "blood flow" OR "flow mediated dilation" OR FMD OR "FMD-BA" OR ultrasound OR acetylcholine OR "nitric oxide" OR microvascular OR microcirculation OR "peripheral artery tonometry" OR "brachial artery reactivity test" OR BART OR "reactive hyperaemia" OR RH-PAT OR EndoPAT) OR advanced_abstract_en:(endothelium OR endothelial OR vascular OR vasodilation OR vasodilator OR "blood flow" OR "flow mediated dilation" OR FMD OR "FMD-BA" OR ultrasound OR acetylcholine OR "nitric oxide" OR microvascular OR microcirculation OR "peripheral artery tonometry" OR "brachial artery reactivity test" OR BART OR "reactive hyperaemia" OR RH-PAT OR EndoPAT)))) [Filters: protocol=no]

**PROSPERO:**

**#1** MeSH DESCRIPTOR Metformin EXPLODE ALL TREES

**#2** endothelium OR endothelial OR vascular OR vasodilation OR vasodilator OR blood flow OR flow mediated dilation OR FMD OR FMD-BA OR ultrasound OR acetylcholine OR nitric oxide OR microvascular OR microcirculation OR peripheral artery tonometry OR brachial artery reactivity test OR BART OR reactive hyperaemia OR RH-PAT OR EndoPAT

**#3** #1 AND #2

**Clinicaltrials.gov:**

*Condition or disease:* endothelium OR endothelial OR vascular OR vasodilation OR “flow mediated dilation” OR FMD OR “nitric oxide” OR microvascular OR “peripheral artery tonometry” OR “brachial artery reactivity test” OR BART OR “reactive hyperaemia” OR RH-PAT OR EndoPAT

*Other terms:* metformin

*Study type:* All Studies

*Study results:* All Studies

**World Health Organization’s International Clinical Trials Registry Platform:**

*Condition:* endothelium OR endothelial OR vascular OR vasodilation OR vasodilator OR “blood flow” OR “flow mediated dilation” OR FMD OR “FMD-BA” OR ultrasound OR acetylcholine OR “nitric oxide” OR microvascular OR microcirculation OR “peripheral artery tonometry” OR “brachial artery reactivity test” OR BART OR “reactive hyperaemia” OR RH-PAT OR EndoPAT

AND

*Intervention:* Metformin in the Intervention

*Recruitment status*: ALL
