# Supplementary material for: Metformin for endothelial dysfunction in non-diabetic disorders: a scoping review
Source: BMJ Open. 2025 Oct 6;15(10):e100017. doi: 10.1136/bmjopen-2025-100017 (PMC12506206; doi:10.1136/bmjopen-2025-100017)
Supplement: online supplemental file 3 [file bmjopen-15-10-s003.pdf]

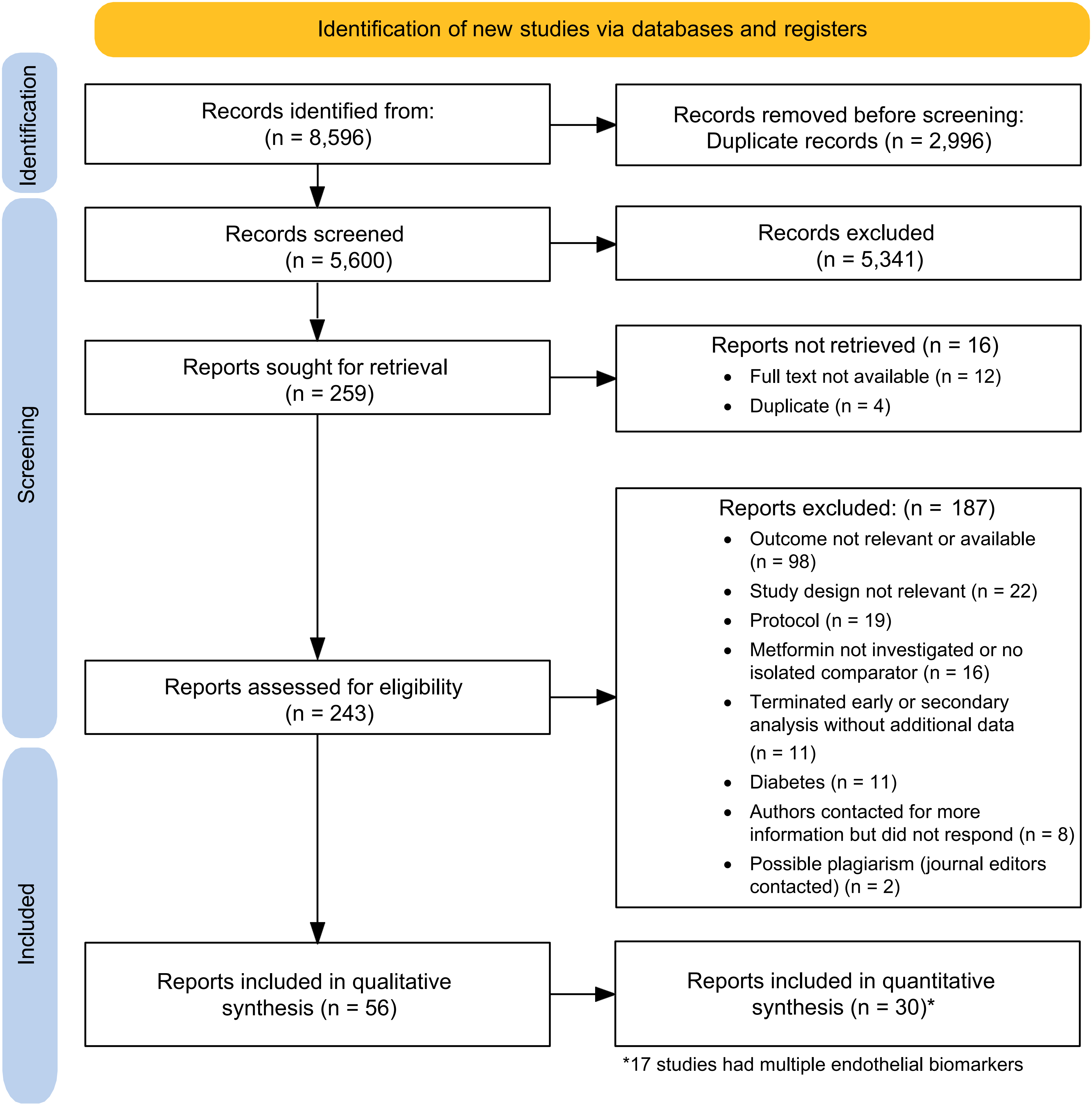
Identification of new studies via databases and registers
Identification
Screening
Included
Records identified from:
(n = 8,596)
Records removed before screening:
Duplicate records (n = 2,996)
Records screened
(n = 5,600)
Records excluded
(n = 5,341)
Reports sought for retrieval
(n = 259)
Reports not retrieved (n = 16)
Full text not available (n = 12)
Duplicate (n = 4)
Reports assessed for eligibility
(n = 243)
Reports excluded: (n = 187)
Outcome not relevant or available (n = 98)
Study design not relevant (n = 22)
Protocol (n = 19)
Metformin not investigated or no isolated comparator (n = 16)
Terminated early or secondary analysis without additional data (n = 11)
Diabetes (n = 11)
Authors contacted for more information but did not respond (n = 8)
Possible plagiarism (journal editors contacted) (n = 2)
Reports included in qualitative synthesis (n = 56)
Reports included in quantitative synthesis (n = 30)*
*17 studies had multiple endothelial biomarkers

- The updated search identified an additional 291 records:
    - Duplicates removed (n = 5)
    - Abstracts excluded (n = 276)
    - Full texts sought (n = 10)
        - Full text excluded (n = 10)
            - Primary studies (n = 7)
                - Outcome not relevant (n = 4)
                - Animal study (n = 1)
                - In vitro study (n = 1)
                - Protocol of excluded study (n = 1)
            - Systematic reviews excluded (n = 3)
                - No additional primary studies
