# Supplementary material for: Metformin for endothelial dysfunction in non-diabetic disorders: a scoping review
Source: BMJ Open. 2025 Oct 6;15(10):e100017. doi: 10.1136/bmjopen-2025-100017 (PMC12506206; doi:10.1136/bmjopen-2025-100017)
Supplement: online supplemental file 5 [file bmjopen-15-10-s005.pdf]

Supplementary 5: HIV studies

Study author	NCD	Number of participants	Time since HIV diagnosis (years)	Plasma HIV viral load (copies/mL)	CD4 count (cells/mL)	ART regimen	Duration on ART (years)
Van Wijk*	HIV lipodystrophy	Metformin arm: 20<br>Rosiglitazone arm: 19	Metformin arm: 7.5 ± 4.0<br>Rosiglitazone arm: 8.4 ± 4.4	Metformin: 50 (50-50)†<br>Rosiglitazone: 50 (50-105)†	Metformin: 574 ± 259<br>Rosiglitazone: 697 ± 366	<u>Metformin:</u><br>- 60% (12/20) on NRTI + PI<br>- 40% (8/20) on NRTI + NNRTI<br><u>Rosiglitazone:</u><br>- 68.4% (13/19) on NRTI + PI<br>- 31.6% (6/19) on NRTI + NNRTI	Metformin: 5.4 ± 3.6<br>Rosiglitazone: 5.6 ± 3.5
Fitch‡	Atherosclerosis in PWH	Metformin arm: 13<br>ILSM arm: 11	Metformin arm: 12 ± 1<br>ILSM arm: 14 ± 2	Metformin: 2.33 ± 0.28¶<br>ILSM: 1.93 ± 0.14¶	Metformin: 406 ± 72<br>ILSM: 691 ± 129	<u>Metformin:</u><br>- 76.9% (10/13) on NRTI<br>- 15.4% (2/13) on NNRTI<br>- 46.2% (6/13) on PI<br><u>ILSM:</u><br>- 81.8% (9/11) on NRTI<br>- 9.1% (1/11) on NNRTI<br>- 54.6% (6/11) on PI	Metformin and ILSM: 6 ± 1

ILSM: intensive lifestyle modification; NNRTI: non-nucleoside reverse transcriptase inhibitor; NRTI: nucleoside reverse transcriptase inhibitor; PI: protease inhibitor

* Data reported as mean ± standard deviation (SD)

†Data reported as median (interquartile range)

‡Data reported as mean ± standard error of the mean (SEM)

¶HIV viral load expressed as log10 copies/mL
