# Supplementary material for: Metformin for endothelial dysfunction in non-diabetic disorders: a scoping review
Source: BMJ Open. 2025 Oct 6;15(10):e100017. doi: 10.1136/bmjopen-2025-100017 (PMC12506206; doi:10.1136/bmjopen-2025-100017)
Supplement: online supplemental file 7 [file bmjopen-15-10-s007.pdf]

Study	Metformin N	Metformin Mean	Metformin SD	Comparator N	Comparator Mean	Comparator SD	Mean diff. with 95% CI	Weight (%)
Kelly (2012)	25	-0.17	0.72	25	0.01	0.68	-0.18 [-0.57, 0.21]	28.97
Javed (2020)	20	-0.10	0.56	19	-0.10	0.62	0.00 [-0.37, 0.37]	31.55
Wong (2012)	39	0.12	0.54	23	0.06	0.70	0.06 [-0.27, 0.39]	39.49

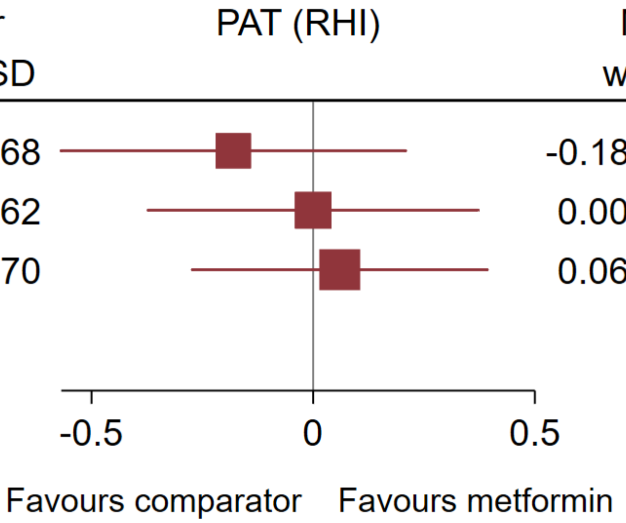

Random-effects REML model
Sorted by: _meta_es

Supplementary Figure 7a: Peripheral arterial tonometry (reactive hyperaemia index)

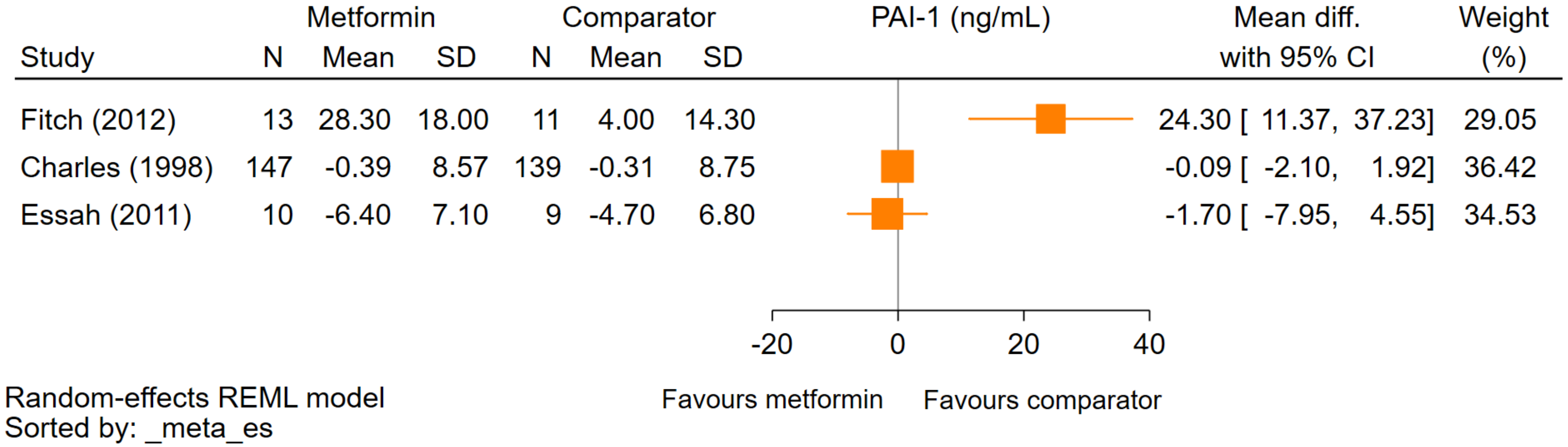

Supplementary Figure 7b: Plasminogen activator inhibitor-1

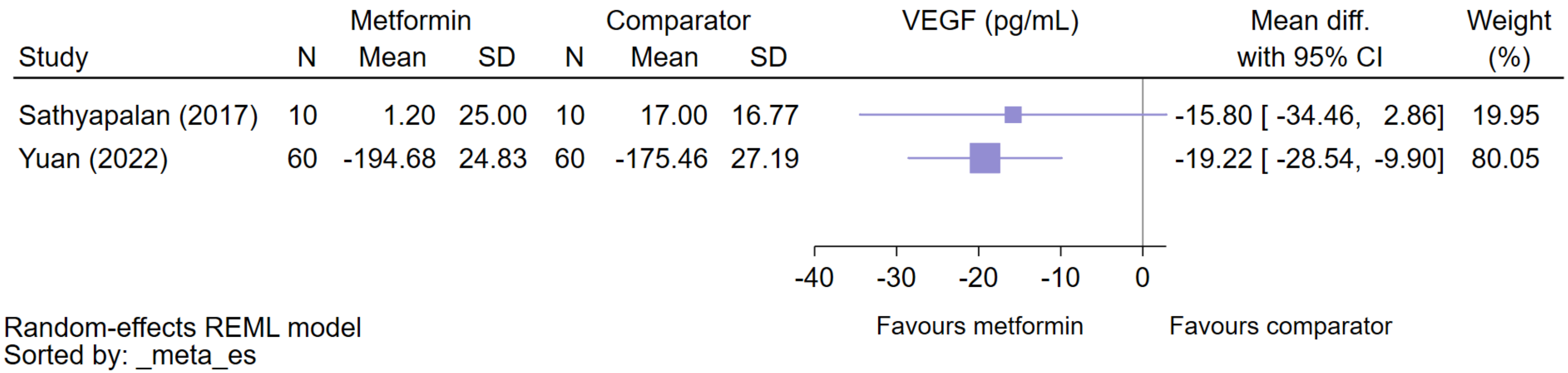

Supplementary Figure 7c: Vascular endothelial growth factor

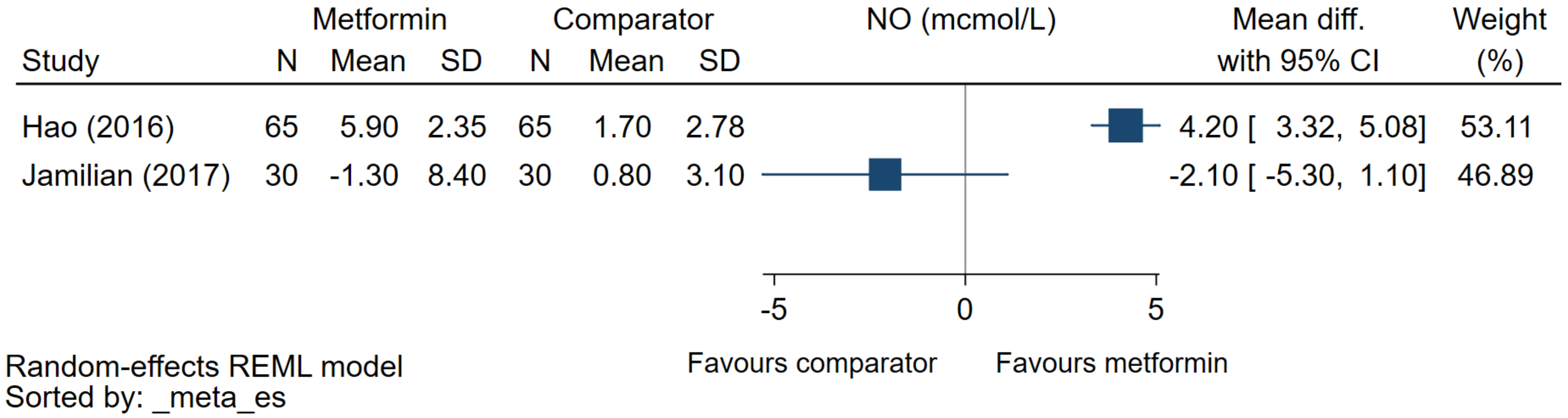

Supplementary Figure 7d: Nitric oxide

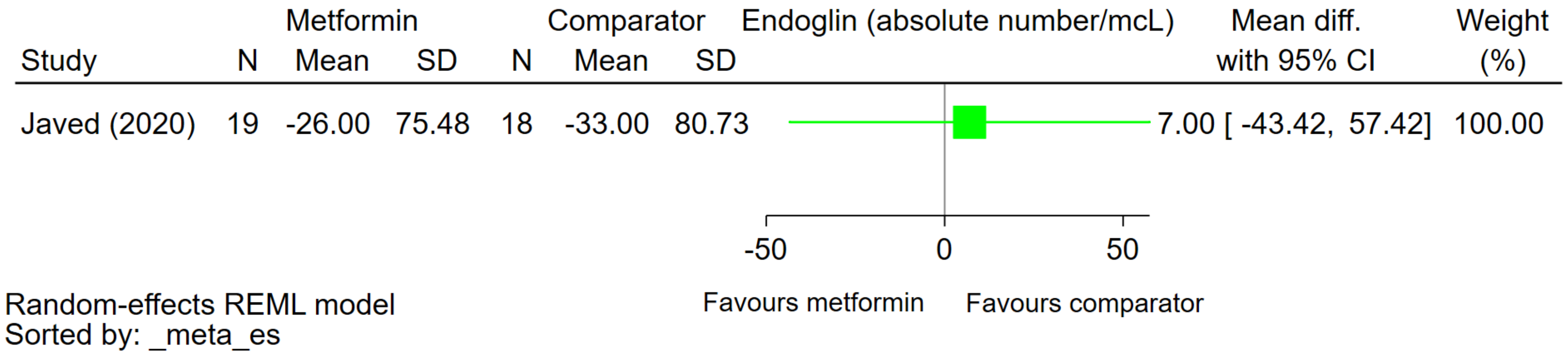

Supplementary Figure 7e: Endoglin

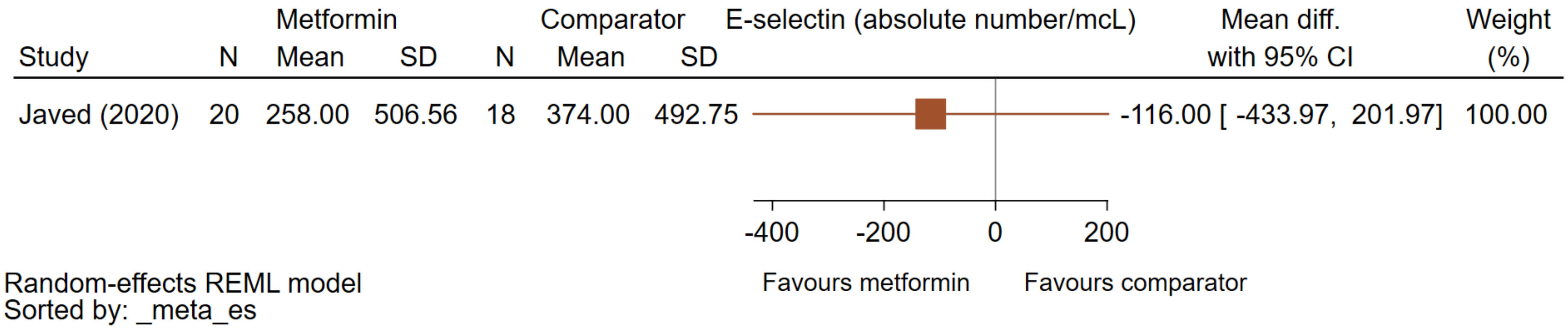

Supplementary Figure 7f: E-selectin

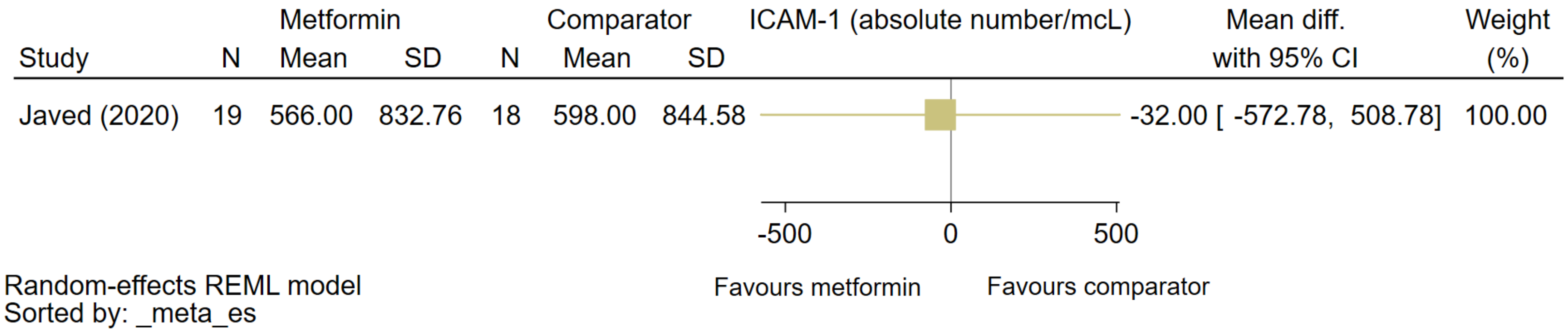

Supplementary Figure 7g: Intercellular adhesion molecule-1

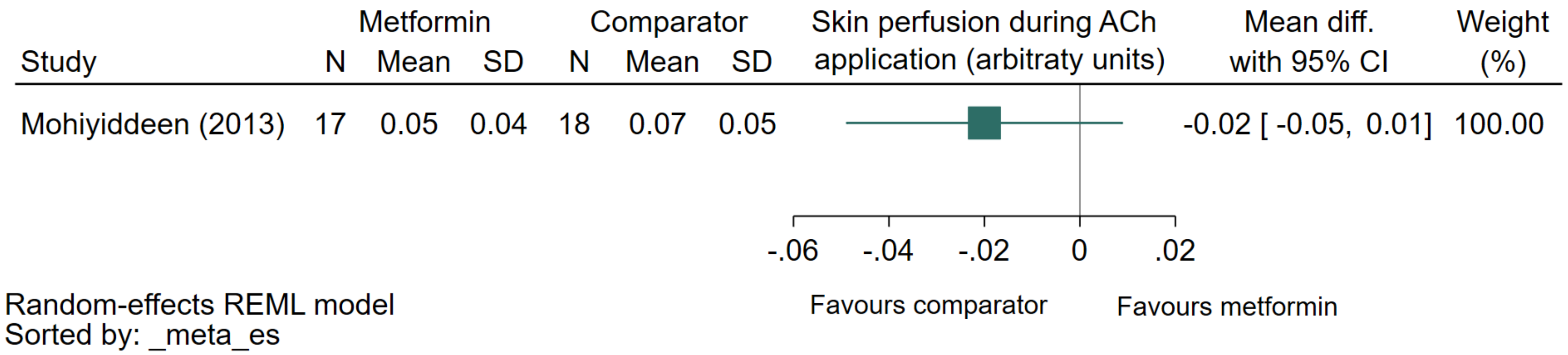

Supplementary Figure 7h: Skin perfusion during acetylcholine application using laser doppler imaging

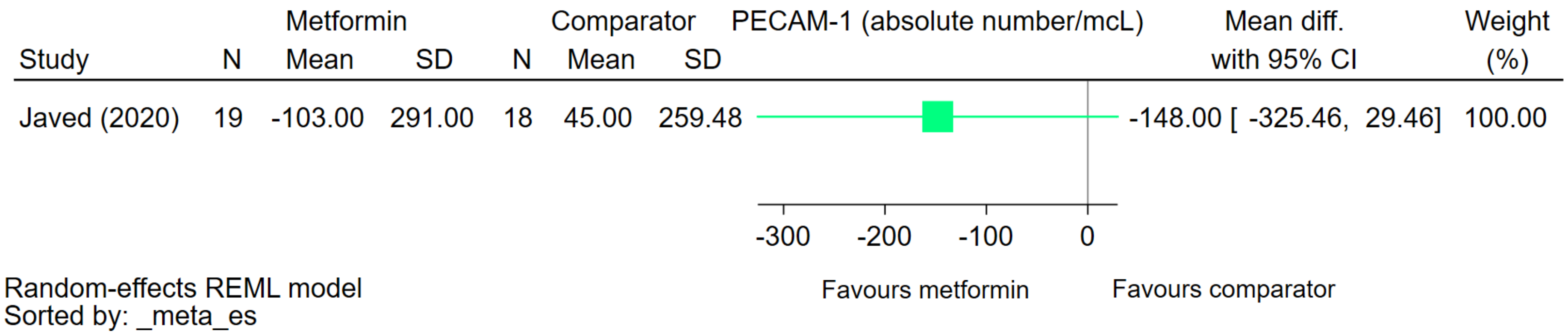

Supplementary Figure 7i: Platelet endothelial cell adhesion molecule-1

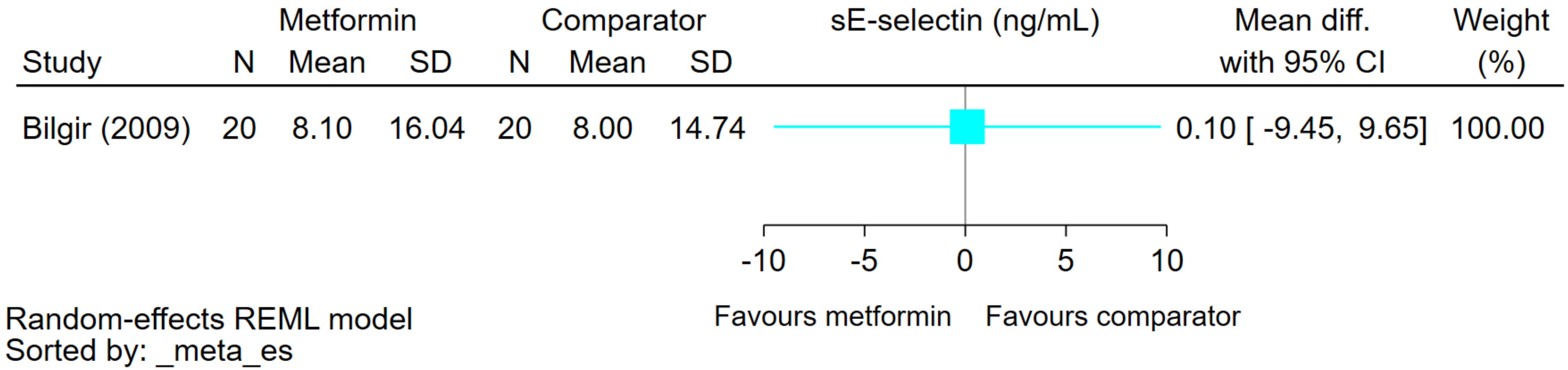

Supplementary Figure 7j: Serum E-selectin

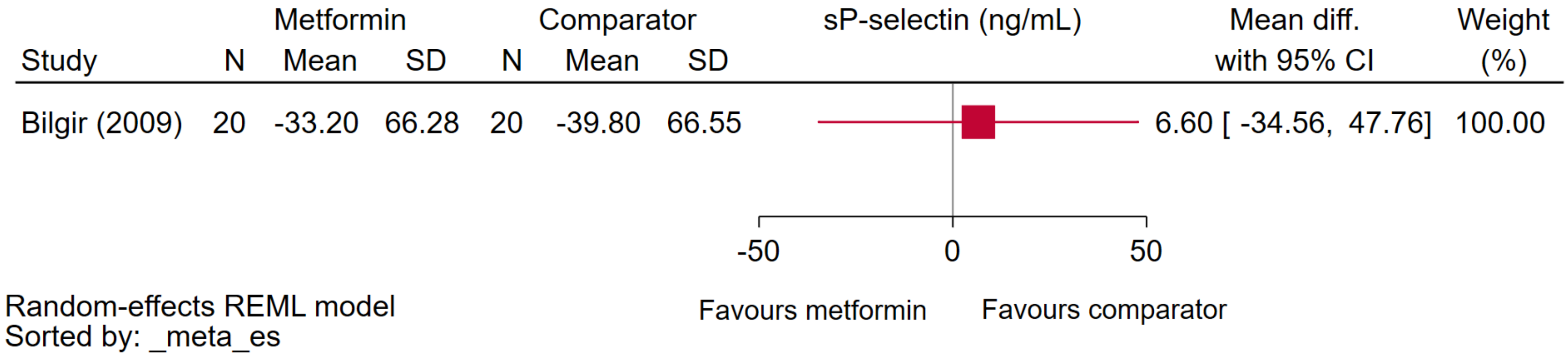

Supplementary Figure 7k: Serum P-selectin

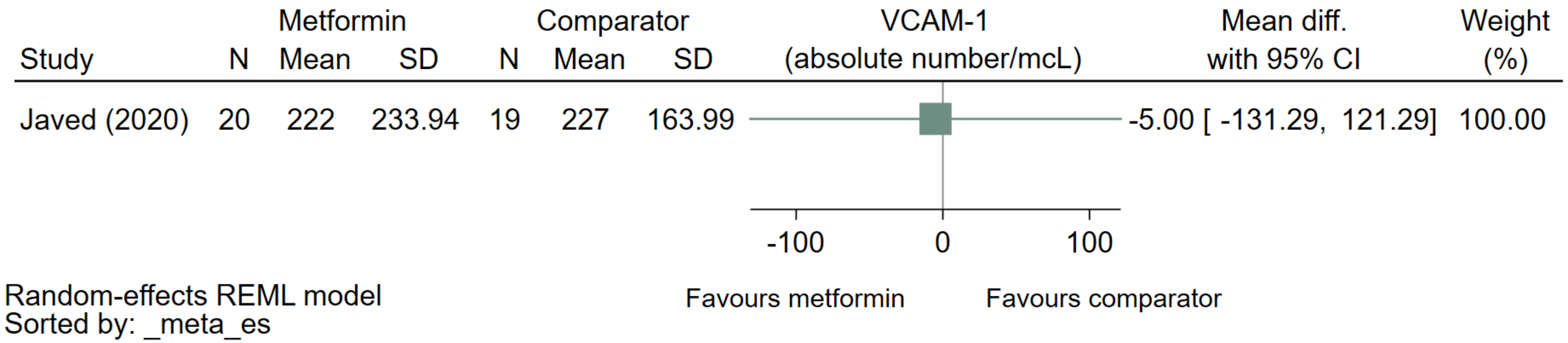

Supplementary Figure 7l: Vascular cell adhesion molecule-1

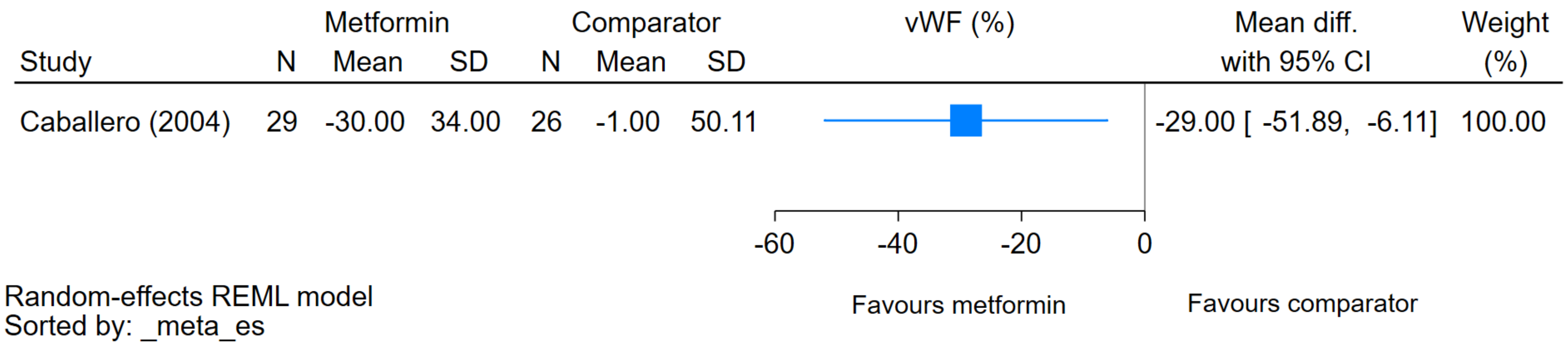

Supplementary Figure 7m: Von Willebrand factor

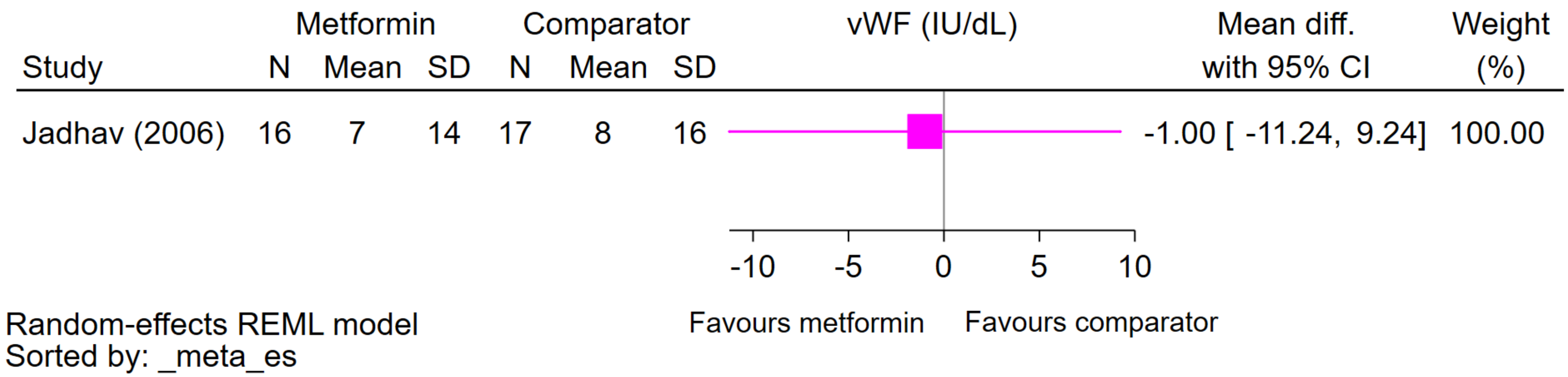

Supplementary Figure 7n: Von Willebrand factor

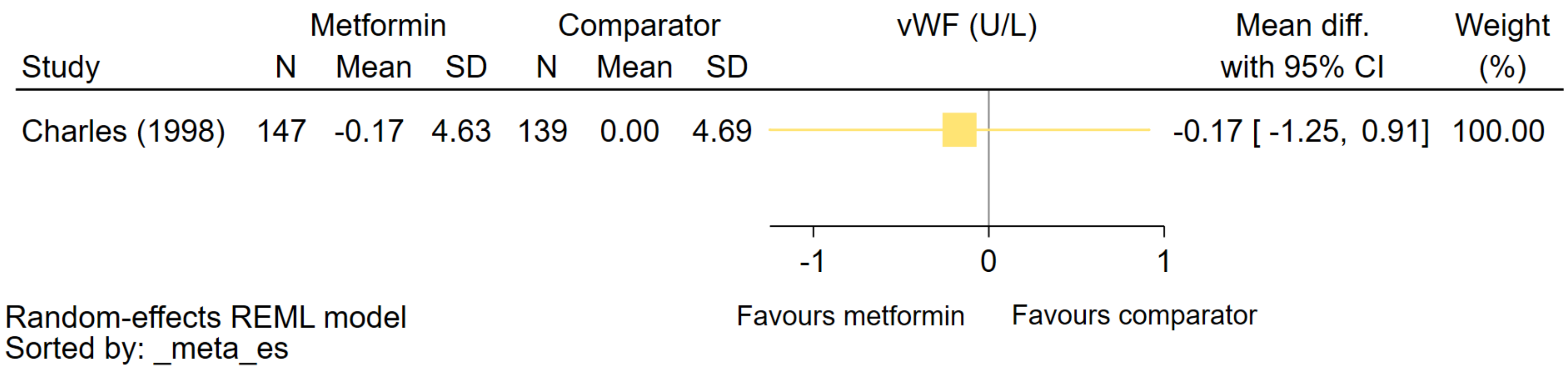

Supplementary Figure 7o: Von Willebrand factor

Supplementary Figure 7: Additional biomarkers showing the mean difference in effect between metformin and the comparator.
