# Supplementary material for: Metformin for endothelial dysfunction in non-diabetic disorders: a scoping review
Source: BMJ Open. 2025 Oct 6;15(10):e100017. doi: 10.1136/bmjopen-2025-100017 (PMC12506206; doi:10.1136/bmjopen-2025-100017)
Supplement: Supplementary file 8 [file bmjopen-2025-100017.reviewer_comments.pdf]

PEER REVIEW HISTORY

BMJ Open publishes all reviews undertaken for accepted manuscripts. Reviewers are asked to complete a checklist review form and are provided with free text boxes to elaborate on their assessment. These free text comments are reproduced below.

ARTICLE DETAILS

Title (Provisional)

Metformin for endothelial dysfunction in non-diabetic disorders: a scoping review

Authors

van Rensburg, Roland; Schoonees, Anel; Ali, Mohammed W; U Van Zyl, Gert; Decloedt, Eric

VERSION 1 - REVIEW

**Reviewer**	**1**
**Name**	**Alizade, Ares**
**Affiliation**	**Van Yuzuncu Yil University**
**Date**	**26-Jun-2025**
**COI**	**None**

This review aimed to map the extent and nature of current research on metformin for endothelial dysfunction in non-diabetic NCDs. This scoping review was conducted in accordance with the methodological framework of Arksey and O'Malley and reported in accordance with the Preferred Reporting Items for Systematic Reviews and Meta-Analyses (PRISMA) extension for scoping reviews.

By evaluating peer-reviewed studies on the use of metformin for endothelial dysfunction in non-diabetic NCDs in adults, as well as excluding narrative reviews, expert opinion, preclinical studies, and qualitative studies only, I believe that valuable information on this topic will contribute to the literature and can be published as such.

**Reviewer**	**2**
**Name**	**Sardu, Celestino**
**Affiliation**	**University of Campania**
**Date**	**16-Jul-2025**
**COI**	**None**

Dear editor,

About the manuscript titled “Metformin for endothelial dysfunction in non-diabetic disorders: a scoping review” enclosed my suggestion:

INTRODUCTION: Provides a clear rationale for the study.

Well contextualized in terms of global NCD burden and endothelial dysfunction.

Justifies repurposing metformin for endothelial dysfunction beyond glycemic control.

While it notes pleiotropic effects of metformin, it lacks detailed molecular mechanisms in non-diabetic contexts.

The rationale for selecting a scoping review over a systematic review is not immediately well justified in this section (though later explained in Methods).

About the metformin, please discuss about the metformin effects on the expression of Sodium-Glucose Cotransporter 2, Leptin, and SIRT6 Levels in Pericoronary Fat Excised from Pre-Diabetic Patients with Acute Myocardial Infarction (Biomedicines. 2021 Jul 28;9(8):904. doi: 10.3390/biomedicines9080904). Thus, the effects of metformin on the CVDs could be explained by significant reduction of inflammatory/oxidative distress (Cardiovasc Diabetol. 2019 Sep 30;18(1):126. doi: 10.1186/s12933-019-0931-0), and amelioration of endothelial function (Diabetes Care. 2019 Oct;42(10):1946-1955. doi: 10.2337/dc18-2356). Please, discuss these points.

Please, discuss about the new-emerging risk factor of CVDs, as body fat accumulation (Front Physiol. 2018 Aug 21;9:1030. doi: 10.3389/fphys.2018.01030), and distribution in particular district as for the breast tissue in the women (JACC Cardiovasc Imaging. 2021 Feb;14(2):426-438. doi: 10.1016/j.jcmg.2020.08.028), and their implication in the MACE at follow-up. The fat tissue accumulation is implied in local and systemic over-inflammation (Front Physiol. 2018 Aug 21;9:1030. doi: 10.3389/fphys.2018.01030; JACC Cardiovasc Imaging. 2021 Feb;14(2):426-438. doi: 10.1016/j.jcmg.2020.08.028) and expression of SGLT2-mediated pathways (Eur J Intern Med. 2023 Jul;113:57-68. doi: 10.1016/j.ejim.2023.04.012. Please, introduce and discuss this point and the suggested references in the text.

Indeed, over-inflammation is a risk factor of CVDs and worse outcomes: Curr Pharm Des. 2020;26(22):2565-2573. doi: 10.2174/1381612826666200213123029. Please, discuss this point and the reference as suggested.

METHODS: Single-reviewer screening for titles/abstracts raises a risk of selection bias, despite the mitigation strategy of overinclusion.

No quality assessment of included studies, which limits the robustness of conclusions regarding metformin's efficacy.

Heterogeneity of study designs (RCTs, pre-post, observational) complicates the interpretation of synthesized data.

Grey literature and non-English studies were not included, potentially introducing publication/language bias.

RESULTS: Heavy concentration of studies on PCOS and metabolic syndrome (88.6% of participants), reducing generalisability.

Only 5 studies (9%) from the global south.

High proportion of studies (64.3%) without active comparators, affecting causal inference.

Only 7 of 19 biomarkers showed a trend toward improvement—limiting conclusions about efficacy.

DISCUSSION: Reliance on pre-post designs and non-blinded studies is noted but could be better emphasized as a source of bias.

Discussion of biomarker limitations (e.g., operator dependence in FMD/CIMT) is insightful but lacks alternative proposals.

The analysis of duration effects on efficacy is inconclusive but speculative—acknowledged yet not deeply probed.

FIGURES AND TABLES: Figures on biomarker effect sizes are visual only without statistical inference, which might be misinterpreted as definitive.

Supplementary materials are referenced frequently but not summarized or included in the main narrative.

Major Limitations of the Study:

Lack of meta-analytic synthesis or effect size estimation.

No critical appraisal of included studies.

Potential selection and detection biases due to non-blinded study designs and single-reviewer initial screening.

Overrepresentation of PCOS and metabolic-endocrine NCDs limits broader applicability.

VERSION 1 - AUTHOR RESPONSE

Reviewers' Comments:

Reviewer #1: This review aimed to map the extent and nature of current research on metformin for endothelial dysfunction in non-diabetic NCDs. This scoping review was conducted in accordance with the methodological framework of Arksey and O'Malley and reported in accordance with the Preferred Reporting Items for Systematic Reviews and Meta-Analyses (PRISMA) extension for scoping reviews.

By evaluating peer-reviewed studies on the use of metformin for endothelial dysfunction in non-diabetic NCDs in adults, as well as excluding narrative reviews, expert opinion, preclinical studies, and qualitative studies only, I believe that valuable information on this topic will contribute to the literature and can be published as such.

We thank the reviewer for their evaluation of the manuscript, and their support of it in its current form.

Reviewer #2: About the manuscript titled "Metformin for endothelial dysfunction in non-diabetic disorders: a scoping review" enclosed my suggestion:

INTRODUCTION:

1. Provides a clear rationale for the study.

2. Well contextualized in terms of global NCD burden and endothelial dysfunction.

3. Justifies repurposing metformin for endothelial dysfunction beyond glycemic control.

4. While it notes pleiotropic effects of metformin, it lacks detailed molecular mechanisms in non-diabetic contexts.

   We thank the reviewer for highlighting this omission. We have added proposed molecular mechanisms to the manuscript, as detailed in comments 6 and 8 below.

5. The rationale for selecting a scoping review over a systematic review is not immediately well justified in this section (though later explained in Methods).

   We thank the reviewer for raising this important point. Prior to conducting our search, the scope and extent of the literature on the research question was unknown, and a scoping review was deemed more appropriate to map out the available evidence, to identify possible gaps (the reviewer noted lack of data from the global south, for example), and to use these findings to better inform a future systematic review with meta-analysis, if the findings lend itself to such. As we found adequate data that can be systematically synthesised, we recommend a systematic review as a subsequent step in the manuscript: "Our review highlights potential research opportunities and provides a platform for conducting future high-quality research… in particular systematic reviews and meta-analyses."

6. About the metformin, please discuss about the metformin effects on the expression of Sodium-Glucose Cotransporter 2, Leptin, and SIRT6 Levels in Pericoronary Fat Excised from Pre-Diabetic Patients with Acute Myocardial Infarction (Biomedicines. 2021 Jul 28;9(8):904. doi: 10.3390/biomedicines9080904). Thus, the effects of metformin on the CVDs could be explained by significant reduction of inflammatory/oxidative distress (Cardiovasc Diabetol. 2019 Sep 30;18(1):126. doi: 10.1186/s12933-019-0931-0), and amelioration of endothelial function (Diabetes Care. 2019 Oct;42(10):1946-1955. doi: 10.2337/dc18-2356). Please, discuss these points.

   We thank the reviewer for the provided citations. We have amended and added the following section to the manuscript with the suggested citations: "Metformin has been shown significantly improve endothelial dysfunction, independent of its glucose-lowering

mechanism.[18] Illustratively, in a study of 172 propensity score–matched participants with prediabetes and stable angina (with half on metformin), those on metformin had less coronary artery endothelial dysfunction as assessed by an acetylcholine infusion than those not on metformin ($p<0.05$).[28] This beneficial outcome is postulated to be linked to metformin's pleiotropic effects to modulate molecular mechanisms, including the reduction of oxidative stress and pro-inflammatory cytokines such as tumour necrosis factor alpha and interleukin-6, and the normalisation of anti-inflammatory biomarkers such as adiponectin and sirtuin-6.[18,29,30]"

While metformin modulating SGLT-2 expression is promising, we focussed on including more widely investigated (plasma) biomarkers, as reported in the above amendment.

7. Please, discuss about the new-emerging risk factor of CVDs, as body fat accumulation (Front Physiol. 2018 Aug 21;9:1030. doi: 10.3389/fphys.2018.01030), and distribution in particular district as for the breast tissue in the women (JACC Cardiovasc Imaging. 2021 Feb;14(2):426-438. doi: 10.1016/j.jcmg.2020.08.028), and their implication in the MACE at follow-up. The fat tissue accumulation is implied in local and systemic over-inflammation (Front Physiol. 2018 Aug 21;9:1030. doi: 10.3389/fphys.2018.01030; JACC Cardiovasc Imaging. 2021 Feb;14(2):426-438. doi: 10.1016/j.jcmg.2020.08.028) and expression of SGLT2-mediated pathways (Eur J Intern Med. 2023 Jul;113:57-68. doi: 10.1016/j.ejim.2023.04.012. Please, introduce and discuss this point and the suggested references in the text.

   We thank the reviewer for the suggested angle. However, overweight/obesity was directly investigated in only 4/56 studies, contributing only 11% of the total included population. Additionally, we did not have data on fat distribution and location, limiting the application of the suggested references to the current scoping review, and potentially detracting from the aim of the review which was to scope the evidence and identify gaps in the literature.

8. Indeed, over-inflammation is a risk factor of CVDs and worse outcomes: Curr Pharm Des. 2020;26(22):2565-2573. doi: 10.2174/1381612826666200213123029. Please, discuss this point and the reference as suggested.
   We have added the reference as per point 6 above.

METHODS:

9. Single-reviewer screening for titles/abstracts raises a risk of selection bias, despite the mitigation strategy of overinclusion.

   We fully acknowledge the limitation of the single-reviewer screening approach. As the noted mitigation strategy favoured overinclusion at the initial stage, it reduced the likelihood of excluding eligible studies prematurely, and is consistent with recommended pragmatic adaptations for scoping reviews where comprehensiveness is prioritised over early exclusion. However, the limitation is noted in the manuscript.

10. No quality assessment of included studies, which limits the robustness of conclusions regarding metformin's efficacy.
We agree with the reviewer, but scoping reviews do not typically include a formal quality assessment or risk of bias assessment of included studies, as is the case with systematic reviews. The proposed subsequent systematic review and meta-analysis will include quality assessment.

11. Heterogeneity of study designs (RCTs, pre-post, observational) complicates the interpretation of synthesized data.
We agree with the reviewer that there was homogeneity among the study designs. However, we only reported what was found in the search according to our research question, and the identified homogeneity further substantiates our initial step to conduct a scoping review instead of a systematic review.

12. Grey literature and non-English studies were not included, potentially introducing publication/language bias.
We did not apply a language filter to our search, but agree that excluding grey literature could potentially have introduced publication bias. We therefore updated the limitations to reflect this: "Last, grey literature was excluded from the search and could have contributed to publication bias; however, the review aimed to map peer-reviewed and indexed evidence to ensure replicability and methodological transparency."

RESULTS:

13. Heavy concentration of studies on PCOS and metabolic syndrome (88.6% of participants), reducing generalisability.

As with point 11 above, we only reported what was found in the search according to our research question, and note in the manuscript that the findings may not be generalisable.

14. Only 5 studies (9%) from the global south.

This important finding supports the study approach as a scoping review, which is designed to identify gaps in the literature for future studies to address. We have specifically highlighted the lack of studies from the global south as a gap in the manuscript.

15. High proportion of studies (64.3%) without active comparators, affecting causal inference.

As with point 11 and 13 above, we only reported what was found in the search according to our research question, and acknowledge in the manuscript that "The paucity of an independent, external comparator group in these studies may have introduced confounding".

16. Only 7 of 19 biomarkers showed a trend toward improvement—limiting conclusions about efficacy.

We thank the reviewer for highlighting this finding. We only reported what was found in the search according to our research question, and did not make claims about efficacy, merely highlighting trends in the biomarkers. In acknowledgement of this, we state in the manuscript that: "Our approach in conducting the research as a scoping review and not a systematic review with methodological quality appraisal limits the strength of this research to draw conclusions on the effect of metformin on biomarkers of endothelial dysfunction."

DISCUSSION:

17. Reliance on pre-post designs and non-blinded studies is noted but could be better emphasized as a source of bias.

    Thank you for the insightful comment. While study design is a potential source of bias, the scoping review did not select for interventional study designs; it only reported the current extent of the evidence related to the research question, and did not make claims on the causality of metformin's effectiveness for endothelial dysfunction.

18. Discussion of biomarker limitations (e.g., operator dependence in FMD/CIMT) is insightful but lacks alternative proposals.

19. The analysis of duration effects on efficacy is inconclusive but speculative—acknowledged yet not deeply probed.

    We thank the reviewer for emphasising points 18 and 19 above, but would like to highlight that the argument for the operator-dependent biomarkers having a trend for more beneficial results and the study durations as a cause of bias (as examples) were stated as possible explanations, not limitations. From the available data, further extrapolation without substantiation might be misinterpreted as evidence of effect, which we want to avoid.

FIGURES AND TABLES:

20. Figures on biomarker effect sizes are visual only without statistical inference, which might be misinterpreted as definitive.

    We omitted the statistical inference specifically to avoid the misinterpretation as highlighted by the reviewer. To address this potential misinterpretation, we state the following in the manuscript regarding the figures: "The overall effect size and heterogeneity statistics were not reported to avoid interpretation as a meta-analysis."

21. Supplementary materials are referenced frequently but not summarized or included in the main narrative.

    We thank the reviewer for identifying this, but we limited more extensive discussion of the supplementary material in the main text as these are complementary to the primary focus of the manuscript, and to be compliant with the journal's requirements.
